# Structural Optimization through Biomimetic-Inspired Material-Specific Application of Plant-Based Natural Fiber-Reinforced Polymer Composites (NFRP) for Future Sustainable Lightweight Architecture

**DOI:** 10.3390/polym12123048

**Published:** 2020-12-19

**Authors:** Timo Sippach, Hanaa Dahy, Kai Uhlig, Benjamin Grisin, Stefan Carosella, Peter Middendorf

**Affiliations:** 1 Integrative Computational Design and Construction (IntCDC), Cluster of Excellence, University of Stuttgart, Keplerstr. 11, 70174 Stuttgart, Germany; ts.sippach@gmail.com; 2 BioMat Department, Bio-Based Materials and Materials Cycles in Architecture, Institute of Building Structures and Structural Design (ITKE), University of Stuttgart, Keplerstr. 11, 70174 Stuttgart, Germany; 3 Department of Architecture (FEDA), Faculty of Engineering, Ain Shams University, Cairo 11517, Egypt; 4 Department of Planning, Technical Faculty of IT & Design, Aalborg University, 2450 Copenhagen, Denmark; 5 Mechanics and Composites Department, Leibniz-Institut für Polymerforschung Dresden e.V., Hohe Str. 6, 01069 Dresden, Germany; uhlig@ipfdd.de; 6 Institute of Aircraft Design (IFB), University of Stuttgart, Pfaffenwaldring 31, 70569 Stuttgart, Germany; grisin@ifb.uni-stuttgart.de (B.G.); stefan.carosella@ifb.uni-stuttgart.de (S.C.); peter.middendorf@ifb.uni-stuttgart.de (P.M.)

**Keywords:** architectural lightweight structure, high-performance structure, biomimetics, topology optimization, material-appropriate design, tailored fiber placement, flax fiber, biocomposites, sustainable architecture, natural fiber reinforced polymer composites NFRP, sustainable architecture

## Abstract

Under normal conditions, the cross-sections of reinforced concrete in classic skeleton construction systems are often only partially loaded. This contributes to non-sustainable construction solutions due to an excess of material use. Novel cross-disciplinary workflows linking architects, engineers, material scientists and manufacturers could offer alternative means for more sustainable architectural applications with extra lightweight solutions. Through material-specific use of plant-based Natural Fiber-Reinforced Polymer Composites (NFRP), also named *Biocomposites*, a high-performance lightweight structure with topology optimized cross-sections has been here developed. The closed life cycle of NFRPs promotes sustainability in construction through energy recovery of the quickly generative biomass-based materials. The cooperative design resulted in a development that were verified through a 1:10 demonstrator, whose fibrous morphology was defined by biomimetically-inspired orthotropic tectonics, generated with by the fiber path optimization software tools, namely *EdoStructure* and *EdoPath* in combination with the appliance of the digital additive manufacturing technique: Tailored Fiber Placement (TFP).

## 1. Introduction

**Figure 1:**
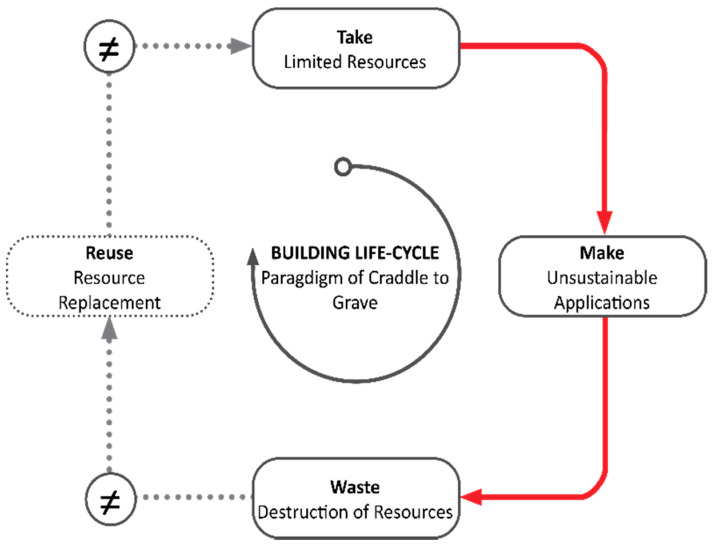
Unsustainable construction ethics.

The constant adaptation to social influences and to environmental constellations are important factors to consider when planning contemporary architecture. Man-made influences on building culture have always been passively influenced by climatic environmental conditions. As a result, the architectural language of form is constantly evolving, and innovative design processes are developed. The cradle to grave paradigm, which is often applied by the building industry, is based on a raw material consumption of “take, make and waste” [1]. In Europe, only 50% of the raw materials used by the construction industry are recyclable [2]. On a global scale, the construction sector is responsible for 36% of the world’s energy consumption and for almost 45% of the world’s resource consumption. The usage of conventional raw materials such as steel, aluminum, and concrete causes an enormous CO_2_ emission of almost 40%, of which 8% is due to the usage of cement [3,4,5,6] (Figure 1). 

**Figure 2:**
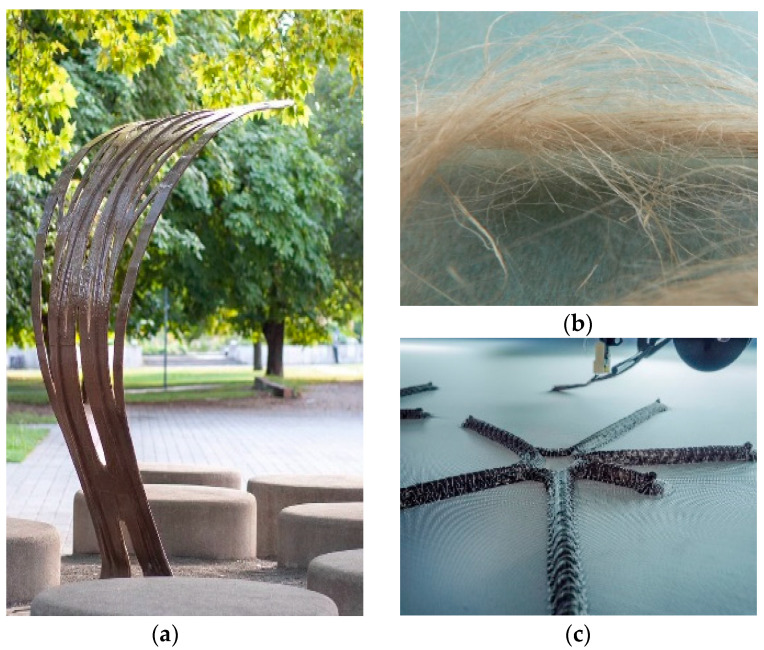
Applications of Tailored Fiber Placement (TFP): (**a**) Tailored Biocomposite Mock-up 2019, BioMat, at ITKE, University of Stuttgart; Single-curved lightweight structure, of 225 cm high and 125 cm width [7]. (**b**) Bango sound distribution element, Institute of Aircraft Design (IFB), University of Stuttgart; Material and geometry distribute sound waves over various surfaces [17]. (**c**) Flax fibers are shown, being the first leading industrial natural fiber in the automotive industry applied in two thirds of the overall natural fiber-reinforced polymer (NFRP) applied in this industry [18].

The resulting change in global climatic conditions is forcing architects to develop new approaches of design thinking. The combination of innovative materials, bio-based engineering principles and new fabrication techniques is a promising way to ensure more sustainable construction methods. The architectural use of polymer composites has a great potential for optimizing lightweight, structural and non-structural applications such as canopies, pavilions, or facade elements (Figure 2) [7,8,9,10,11,12,13,14,15,16].

The possibility of custom configuration of composite components enables the creation of tailor-made material compositions. The adaptation of the fiber orientation in load-bearing geometries according to applied load cases leads to extraordinary performance qualities. The sustainability in structural end-use applications increases through biological building materials. Natural fiber-reinforced polymer composites (NFRP) are already well established in automotive and aircraft industry. They represent a promising solution for future architectural applications.

The integration of biomimetic principles, such as topology optimization enables innovative architectural lightweight constructions. This principle was here applied as a reference for load-bearing structures distributing material paths according to the axiom of uniform stress, which is of great importance and relevance in this project. In addition, the use of well-established fabrication techniques from the aerospace industry can help to revolutionize architectural design, which was as well approached. 

The presented work is the result of close cooperation between architects and engineers in the fields of architecture, aircraft construction and polymer research. The workflow of knowledge and expertise exchange as well as the close collaboration of the research fields are major factors in the development of the project. The use of NFRPs, combined with the application of computational design and digital optimization techniques, were decisive for the successful realization of the architectural lightweight structure through state-of-the-art digital additive fabrication techniques.

### Case Study 

The project’s core is the material-appropriate application of a recyclable natural fiber-reinforced polymer composites to a load-bearing structure using the methods of topology optimization and fiber orientation according to the occurring principal stress trajectories. The outcome highlights the design-related potential of analyzing the properties and restrictions of biological role models, fiber composites and the combination of digital and physical manufacturing methods to find innovative and sustainable architectural lightweight solutions. The segmental design bypasses the size restrictions of TFP production and transfers the use of load-optimized thermoset plant-based NFRP composites produced by TFP to an architectural scale. The fiber path orientation according to the principal stress directions in continuous fiber-reinforced plastics minimizes resource consumption and maximizes load carrying capacity.

The 4 m high and 4 m wide supporting structure has a total volume of 2.15 m^3^ and consists of four identical, double-curved branching elements. They are rotationally symmetric arranged and connected centrally by additional filament winding. The element can be used as a fixed or temporary load-bearing structure for street furniture, facade parts, or as an installation for events and museums. 

Its morphology is derived from a generative topology optimization process consisting of parameter-driven design and optimization simulations (Rhinoceros 3D, Grasshopper, Fusion 360, ANSYS). Secondary manual design adjustments create a load-optimized and material-efficient geometrical design. The structural design and Finite Element (FE) modelling of the variable axial fiber patterns were carried out using two software tools developed for this purpose (EDOPath, EDOStructure). The computational approach enabled the division of the four branches of the structure into six laminate layers. They consist of individual load-optimized variable-axial fiber patterns manufactured using TFP technology. Due to the relatively high strength related to its density, a natural fiber-reinforced polymer (NFRP) based on flax fibers was chosen. The laminates were fabricated using a Tajima multi-head TFP machine type TLMX-T02 and then finished into their final form using resin infusion process and vacuum pressing (VARTM) [7]. Due to given restrictions of the machining areas of the fabrication technology, a demonstrator in scale 1:10 was built.

## 2. Materials and Methods 

**Figure 3:**
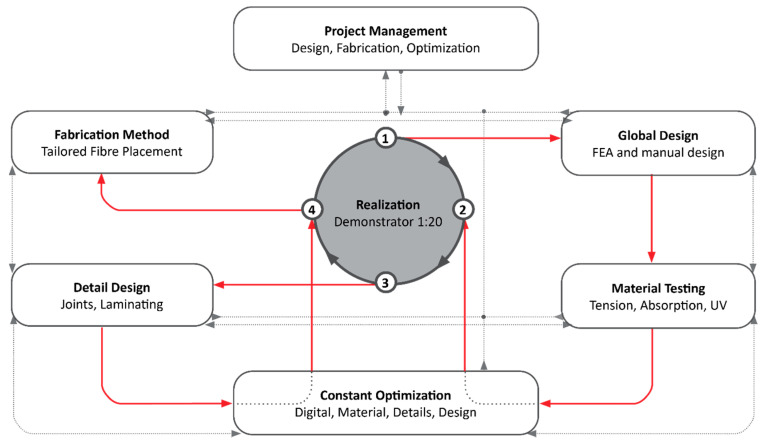
Demonstration of the applied interdisciplinary workflow.

The project started following the design philosophy: “Materials as a Design Tool” [19] combined with a synthesis of primarily inductive and partially deductive methodology. The aim was to further investigate the applicability of Tailored Fiber Placement in the field of architecture and how the material-appropriate application of NFRP can contribute to structural optimization and increase the sustainability level as once indicated using another material development in architecture from the same BioMat group [11]. The iterative design process included manual and digital computational design, parameter driven optimization and digital fabrication. In that sense, the analysis of the biomimetic topology optimization, the modification of specific material properties and the application potentials of additive fabrication technologies were applied. For the constant optimization of the workflows, a continuous exchange of knowledge between the participants took place (Figure 3). The final prototype was evaluated through simulations and physical performance tests, then modified accordingly and constructed as a 1:10 demonstrator.

### 2.1. Analysis Criteria

To evaluate the obtained results of the prototype construction, functional and performative design criteria were restricted to three design parameters:

1. Structural optimization and material reduction through fiber orientation;
2. Material-appropriate application of NFRP for time-efficient fabrication;
3. Development of a demonstrator with a minimum load capacity of 2.000 N.

These criteria have been tested, optimized, and evaluated as inputs in the engineering process. The following sections explain the results in detail to confirm the hypothesis.

The overall design objective of the presented work is the realization of an innovative material-minimizing and performance-maximizing architectural load-bearing structure and its exploration of fabrication-related geometric tolerances, materialized from recyclable thermoset NFRPs. Through the applied methodologies, topological optimization of the structure, appropriate application of customized material properties and combination of the TFP fabrication followed by filament winding was applied. The close cooperation between the engineers of the Leibniz Institute of Polymer Research Dresden e. V., the Institute for Aircraft Design, and the architects of the BioMat Department (Bio-based Materials and Materials Cycles in Architecture) located at the Institute of Building Structures and Structural Design (ITKE) at the University of Stuttgart did not only establish a novel circular design workflow, rather also provided valuable insights within the respective expertise. By means of the intelligent cross-linking of material-appropriate design (orthotropic material properties), digital optimization processes (topology optimization) and scalable design (segmental elements), the project took a cutting-edge role in the development of novel material-saving, high-performance and environmentally friendly load-bearing structures, which was the set hypothesis, then validated through the 1:10 mock-up.

### 2.2. Natural Fiber-Reinforced Polymer Composites (NFRP)

Flax fibers about 2.5–6 cm long were applied that have been joint to 50–90-cm-long yarns through textile engineers and processed into continuous fiber rovings prior to our application [18,20]. Flax fibers were chosen, due to the global increase of its economic and ecologic importance, their CO_2_ neutral life cycle and its relatively low energy consumption in production processes [21,22]. Within the presented work a 1000-Tex flax fiber roving was used (distributor, Group Depestele, Bourguebus, France). The applied thermoset matrix system as provided from Hexion Stuttgart GmbH was EPIKOTE Resin RIMR 135 and the curing agent was EPICURE RIMH 137 [23].

The fiber placement paths mimicked the fibrous structure of plants, which dissipated loads anisotropically along their principal stress axes [24]. Strength, stiffness and flexibility in relation to density of flax fibers are comparable with industrial man-made endless fibers, e.g., glass fibers [25,26]. A possible end-of-life scenario is the grinding of the NFRP and its reintroduction into manufacturing processes. With the possibility of thermal recycling via incineration, energy costs can be partially regained. The high anisotropy and the resulting load-efficient qualities allowed the applied materials to be suitable for architectural freeform structures. In addition, the polymer-encapsulated fibers have already stored CO_2_ during growing before being applied in the material technical cycle as a building material. 

### 2.3. Biomimetics and Topology Optimization

Biomimetics is a growing science branch in which diverse investigations and scientific developments take place including studying the properties of evolutionary proven functions, proportions and patterns of natural systems. It offers an inspiring framework that allows the development of new conceptual designs and the integration in innovative manufacturing techniques. Novel materials and experimental construction principles can be applied into architectural applications through a further technical optimization [27,28,29].

**Figure 4:**
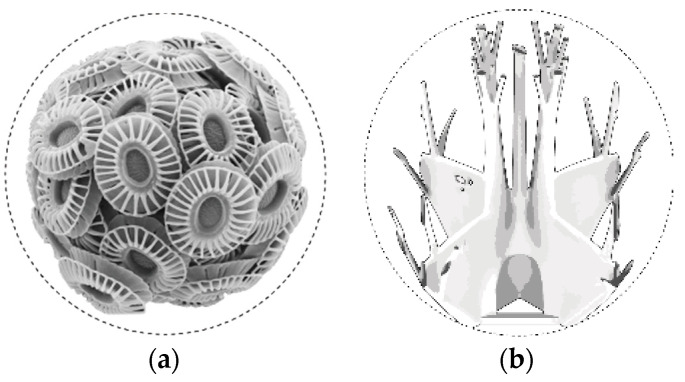
Biological and computational structural optimization. (**a**) Phytoplankton Emiliania Huxley, 10 µm, marine calcite organism composed of a series of coccoliths [35]; (**b**) Topologic optimized design proposal.

In the project, a unicellular microplankton was chosen as the biological role model, from which technical solutions like setting the optimized fiber paths have been “mimicked” and abstracted. The microorganisms that are composed of modular components, so-called coccoliths, have special geometries of individual particles that are determined by environmental conditions such as protective functions and stresses [30,31]. The principle of topology optimization allows a minimal material distribution. This concept was abstracted and applied in the project (Figure 4).

The principle of topology optimization is the central link between design, biomimicry and digital form finding processes. It enabled the creation of more stable and lighter supporting structures. The parameter-determined design process enabled the generation of automated form finding based on the axiom of uniform stress. This FEM process applies boundary conditions such as material properties, loads, supports and restrictions based on a defined domain. In iterative steps solution-oriented geometries are generated, following the “axiom of uniform stress” [32,33,34]. The enormous morphological diversity of the geometry proposals was evaluated and used design-orientated as a creative foundation for the project’s structural composition.

#### Topology Optimization

The topology-optimized design consisted of four identical elements, which together formed a double-symmetrical structure. To determine the global geometry, load-specific fiber orientation, advantages of FEM and further manual design adjustments were applied. Assigning the following parameters were the baseline conditions that were set for the simulative form finding process: The domain (solid 3-D hexagonal solid, 4 × 4 × 4 m, with geometric restriction areas); the supports and loads (principal stress axis generation); materials of investigation (epoxy resin, MDF, aluminum, steel) (Table 1).

**Figure 5:**
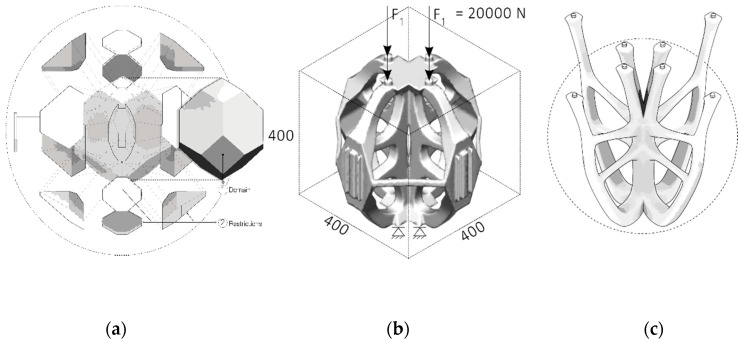
Topology optimization workflow: (**a**) initial domain design; (**b**) parametric form-finding with FEM on restricted domain; (**c**) manual re-design of optimized geometry.

The workflow was divided into three main design phases:

1. Phase: Generative design—Material investigations and FEM-design of draft shape concepts—(Commercial software used: Autodesk Fusion 360 + material library, T-Splines) (Figure 5);
2. Phase: Fiber Design—FEM to generate orthotropic, load-specific fiber paths and laminates; geometry adjustment through thickness distribution dependent on fiber patterns; automatic mold generation. (Software used: EdoStructure, EdoPath developed by IPF);
3. Phase 3: Final Design—Fabrication and visually dependent fiber path and mold adaptation. (Software used: Rhinoceros 3D, T-Splines, Adobe Illustrator).

### 2.4. Design and Manufacturing Process

#### 2.4.1. Tailored Fiber Placement

**Figure 6:**
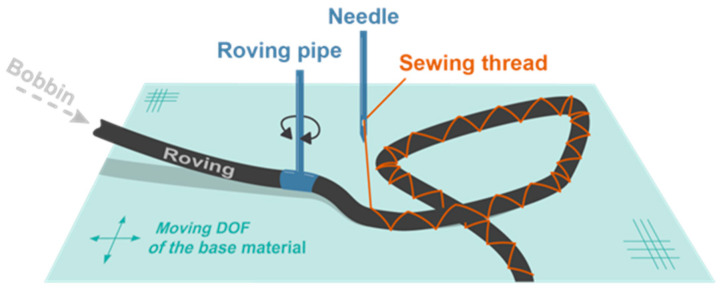
Basic principle of the TFP process.

Tailored Fiber Placement (TFP) is an embroidery-based preform additive manufacturing technique that allows a flexible orientation of any fiber roving (e.g., carbon, glass, aramid). The TFP technology was invented at the Leibniz-Institut für Polymerforschung Dresden e. V. [36]. With this technology a continuous roving is placed along programmable curves within the plane (2D) and fixated by a stitching yarn onto a flat textile base material using a double locked stitch in a zig-zag stitch pattern (Figure 6). The roving is deposited following a pre-defined path by rotating the roving pipe and moving the base material in two perpendicular directions [37]. Due to the technological degrees of freedom and the relatively high placement speed of up to 5 m/min per head, TFP is well suited for producing variable-axial (VA) composites which are also known as variable-stiffness (VS) and variable angle-tow (VAT) composites [38]. 

#### 2.4.2. Fiber Path Pattern Generation

Form finding using Tailored Fiber Placement enables the production of precisely tailored preforms. The automatic preform generation economizes material consumption and minimizes the production of unusable waste materials compared to established Fiber processing techniques. The capability of the TFP technology to produce preforms based on continuous roving materials was assessed by Mattheij et al. [34]. The application of TFP to produce preforms with a curvilinear Fiber pattern were investigated on open-hole tension plates by Crothers et al. [38], Gliesche et al. [39], Temmen et al. [40] and Aschenbrenner et al. [41], and Spickenheuer et al. [42]. Spickenheuer et al. developed a design tool to numerically derive fiber paths along principal stress directions [42]. Spickenheuer et al. [43,44] and Albers et al. [45] combined topology optimization with an appropriate fiber design for producing TFP-based structures. Uhlig et al. [36] developed a parametrical three-dimensional representative unit cell (RUC) based on Finite Elements of TFP based FRP. The representative RUC reflects the TFP specific morphology and allows a better estimation of the resulting material properties depending on the applied TFP parameter. Uhlig et al. [37] experimentally investigated the influence of Tailored Fiber Placement (TFP) processing-related parameters on in-plane waviness and fiber volume content of Unidirectional Carbon Fiber-Reinforced Plastic (UD-CFRP) Composites. Recently, Bittrich et al. [46] proposed a novel framework for optimizing variable axial composites.

After topology optimization, the geometry was segmented to produce fiber paths in principle from unwrappable single or double curved shell structures. Subsequently, this shell structure was meshed with a Finite Element (FE) software (Ansys). The creation of the fiber paths adapted to the defined load case was performed with the software “EDOStructure”, a tool especially developed for the design of VA or VAT FRP laminates. In this step, the FE-mesh was imported in “EDOStructure” and the Fiber paths were defined according to the principal stress directions. The symmetrical element consisted of two mirrored components. The software generated three laminate layers (substrate layer, 1st principal stress layer, 2nd principal layer) per component with 2–5 mm cross-sections. Each layer has an individual geometry that is optimized for the respective load absorption. The resulting thickness distribution determines the structural integrity, volume, and surface properties of the element.

**Figure 7:**
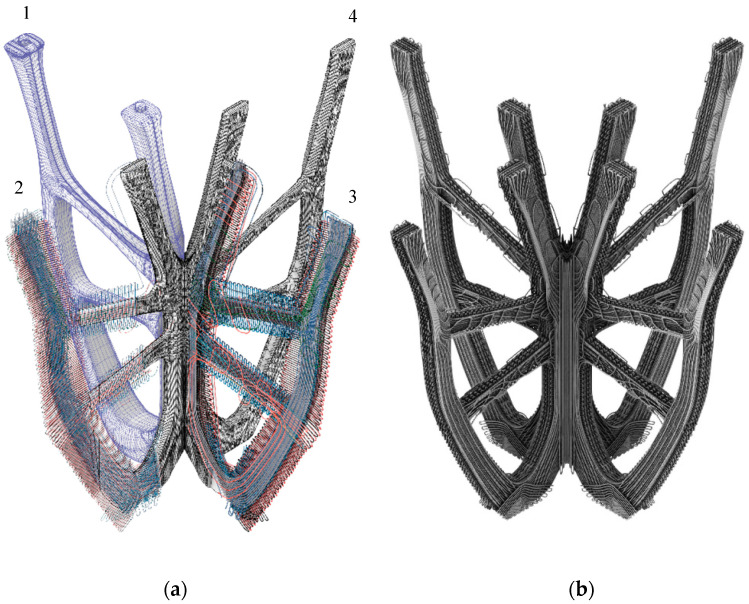
The composition of the load-compatible fiber paths of the geometry. (**a**) Design evolution 1: Concept, 2: Generated pattern, 3: Manual adjustments, 4: Final geometry; (**b**) fiber volume laminate structure.

As the automatically generated fiber patterns did not meet the aesthetic requirements, they were redesigned in Rhinoceros (Figure 7) and re-imported into the “EDOStructure” software. EDOStructure automatically calculates the resulting thickness distribution of the created line pattern based on the used defined material data and takes over the draping process. For an analysis of the component properties EDOStructure creates a 3D FE model with retention of all single layer information. This data set was imported into commercial FE software solutions (Ansys, Autodesk Fusion 360), and the component performance was evaluated in a stress and strain analysis after applying the appropriate boundary conditions and correct material data. When the component properties meet the requirements in the FEA prior to the production of the preform, the generation of the stitch data set for the TFP device is required. Therefore, the software EdoPath was used in this work. EdoPath creates the stitch data automatically based on the Fiber pattern as a *.dxf file. Among others, the average stitch width and stitch distance for each individual layer of the Fiber pattern was set.

#### 2.4.3. Physical Manufacturing Process

**Figure 8:**
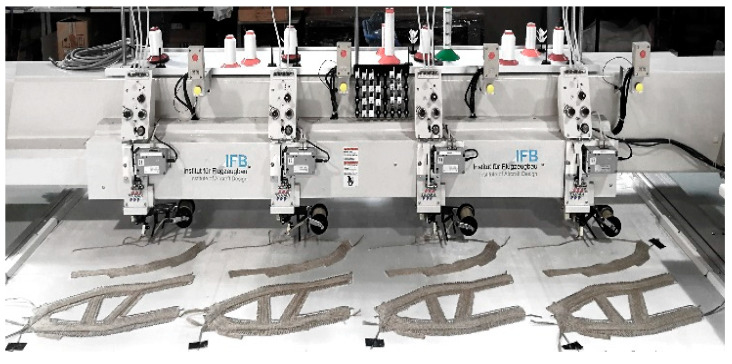
Fabrication with 4-head embroidery Tajima TFP machine, provided by IFB.

The manufacturing of the preform was done with the following process parameters (Table 2). A Tajima 4 head TFP machine Type TLMX-T02 has been used (Figure 8). The machining of the used flax roving did not show any problems or collisions. 

There was no loosing of stitching or failures like blocking of the feeding bobbin. A higher stiffness in the material compared to glass fiber could be observed. It can also be stated that the maximum preform thickness of 10mm of the TFP process has not been reached. The maximum thickness of preforms is 5 mm (sub- preform No.3) or less. To analyze the maximum thickness which can be reached with the used flax material, further trials are necessary in future work.

#### 2.4.4. Vacuum Infusion

After TFP production and trimming of the residual substrate textile, the preforms were infiltrated by means of the Vacuum Assisted Resin Infusion (VARI). For this purpose, commercially available polyethylene vacuum foils were cut as needed and sealed using a vacuum pump (Model P3 from R&G Faserverbundwerkstoffe, max. 55 l/min. at 0.900 bar). The resin compound used, with a mixing ratio of 100:30, was EPIKOTE Resin MGS RIMR 135 with EPIKURE Hardener RIMH 137, manufactured by Hexion Inc. The fiber volume ratio of the preforms consists of 30/70, which required an optimum quantity of approx. 130 g resin per preform (p. P) (Table 3). 

**Figure 9:**
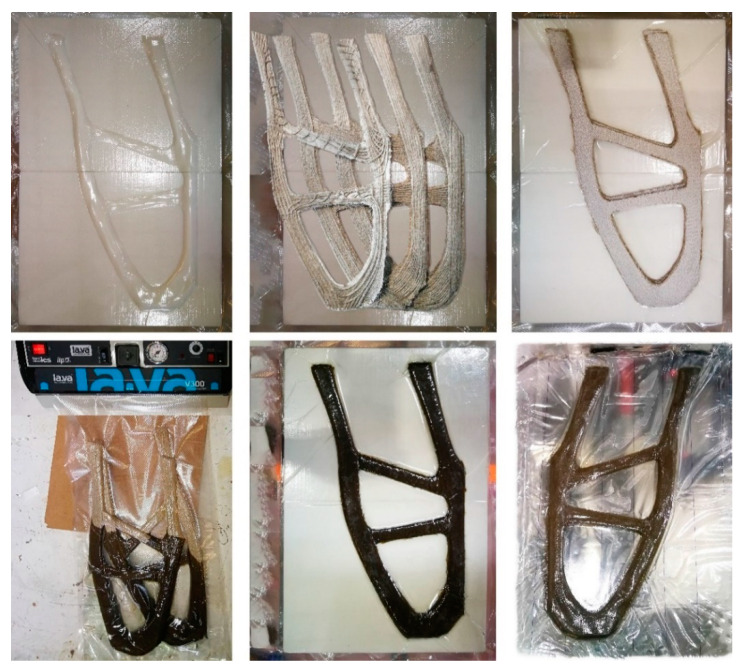
Mold and laminate preparation using airtight sealing bags, resin infusion and vacuuming 292 of stapled laminate layers, demolding at IFB (counterclockwise from top left to bottom right).

To prevent the formation of air bubbles and ensure complete infiltration, 165 g p.P. were used. During the compression process of the resin-impregnated preform, about 30 g of excess resin escaped, resulting in an average weight of 218 g per cured preform. The total manufacturing time of one component is approx. 30 min, with a subsequent curing time of 48 h at room temperature (Figure 9). The table present the results obtained.

#### 2.4.5. Assembly

The designed supporting structure consisted of 4 double-symmetrical elements. A Plexiglas board was fixed on its top and the whole demonstrator was anchored in a resin foundation. Each element consisted of two mirrored preforms, which were sanded along their mirror side, glued, and pressed for 30 min. The 45° angle cut of the preform middle edge enabled a symmetrical composition of the four elements at their centers. All elements were reinforced by an adhesive connection and an additional fiber winding. 

## 3. Results

### Structural Characteristics and Performance 

**Figure 10:**
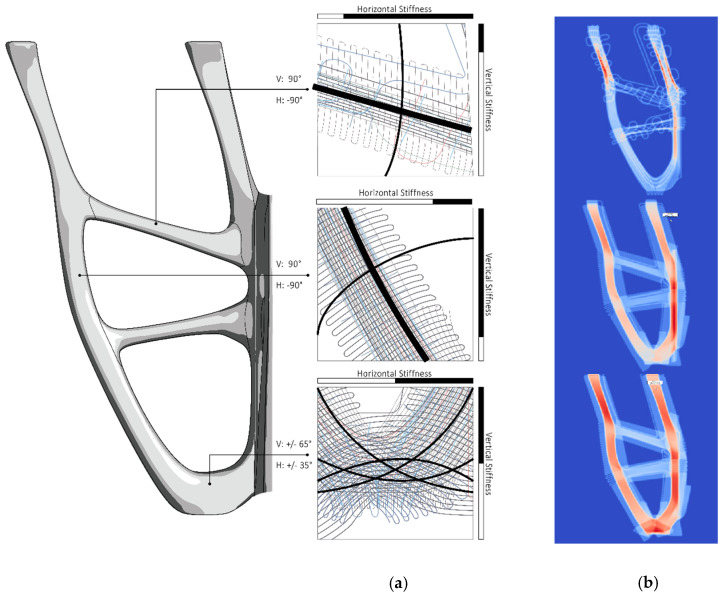
Final geometry—Form follows force: (**a**) Optimized TFP-Pattern for load absorption; (**b**) three laminates with load depending thickness contribution.

After curing and installation of the support panels and foundation, the final mockup was reached. The external dimensions of the overall structure are 65 × 65 cm width and 47 cm height (±0.5 cm). The dimensions of the four branches of the load-bearing structure are 40 × 40 × 40 cm (±0.5 cm) and have a weight of 1744 g (Table 4). The properties of the orthotropic material were optimally exploited and the cross arrangement of the fibers within the associated layers at load-specific angles ensured the structural integrity (Figure 10). The analytical focus of the presented project, as mentioned above, was to determine the material consumption by optimum fiber placement, making good use of the selected digital fabrication method and the applied computational tools simulating structural loads. To stand its own weight, the built demonstrator was targeted to withstand a minimum of 2000 N (200 kg) as a general range for validation of the set hypothesis.

Currently one prototype has been completely manufactured and is used for educational exhibition purposes. Another prototype and further individual custom-made prototypes applying the same concept are still under construction for higher validation of load scenarios. The following steps will include performing 1:1 mechanical tests with the new prototypes, in addition to step-by-step load placement on the carrier plate to evaluate the simulated results under real loading conditions. This series of mechanical tests on standardized specimens will investigate the potential of the structure to withstand much higher loads.

## 4. Discussion

The aim of the project was, on the one hand, to determine the potential for performance enhancement of architectural lightweight structures by material-appropriate application of plant-based natural fibers applied as a reinforcing agent within a biocomposite component using biomimetic topology-optimization scenarios. On the other hand, such developments highlight further possibilities of applying quick renewable resources in the building industry to reduce the construction ecological footprint using additive manufacturing techniques like the chosen tailored fabrication by Tailored Fiber Placement (TFP), adapted from automotive and aircraft design industries to the building construction one. The accomplished combination of methods demonstrates an ultra-lightweight load-bearing mock-up structure capable of bearing approximately 200 times its own weight. The innovative workflow and the interdisciplinary work division, as well as the use of innovative digital fabrication techniques and bio-based materials, is a considerable inspiration for future sustainable architectural practices and engineering design approaches. These workflows establish a novel possibility that contributes to the performance increase of supporting structures by adapting material tectonics. This consists of an intersectional design process of digital and physical optimization, e.g., generation of orthotropic materials or tailoring of preforms.

**Figure 11:**
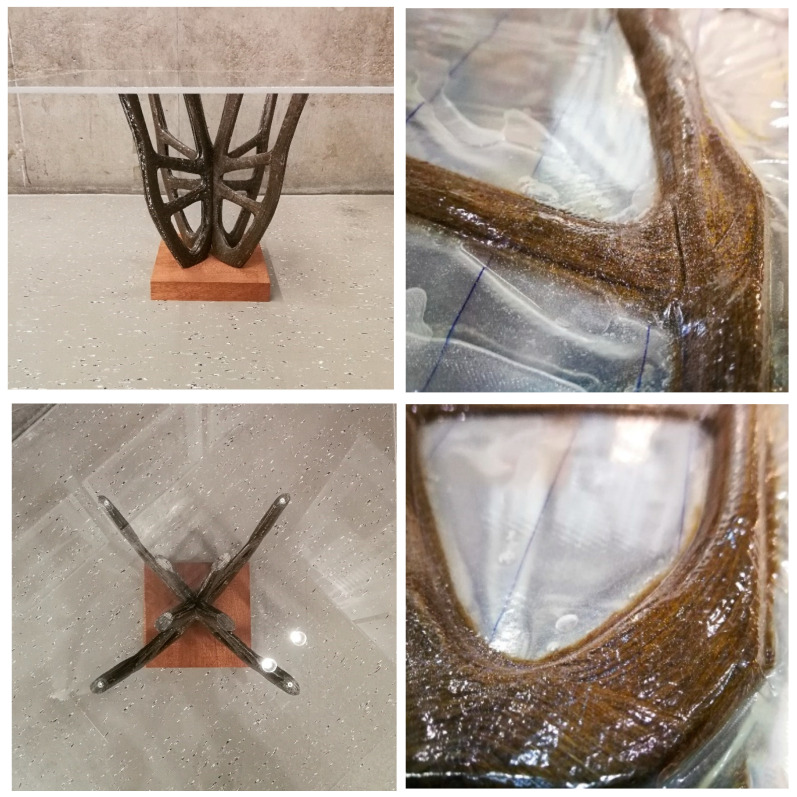
Shape and laminate composition of the structure, close-up of the surface characteristics (counterclockwise from top left to bottom right).

Embroidered on a glass fiber substrate, TFP preforms are given their characteristic topology after shape cutting, resin injection and compression molding to maximize load bearing capacity and structural integrity. The stress related thickness distribution of the fibrous elements and the surface patterns create objects with a natural visual richness. The morphology recalls a classic dendrological structure found in tree branches or root cross-sections and transforms it into new material-specific design expressions (Figure 11).

The relatively short development time of approx. five months did not allow the collection of all further relevant data sets. The obtained data (structural performance, stiffness, material properties, digital and physical optimization processes, etc.) are prototype related. Due to the lack of test series and manual post-processing, this outcome cannot be considered as a fully accurate database for scientific applications by NFRP and TFP. The results should rather be seen as an academic guideline and proof for other means towards designing high-performance sustainable structures using diverse digital approaches and systems responsive to our digital era. Generally, it can be stated, that the knowledge obtained from this development provides satisfactory results that can be used as a guideline for potential sequel projects. In addition, these projects could benefit from a bigger integration of interdisciplinarity with a focus on automation of assembly processes and adaptation of the building materials. Automation processes are already being investigated to bypass time-consuming manual operations. This does not only reduce production time but also improves performance through more precise and repetitive workflows. In this context, it is conceivable to replace conventional fiber rovings with prepreg-rovings. 

Further weight reduction and larger structures could be designed with more sophisticated modular segments. Specialized roving modifications, fiber path layouts and alternative matrices could contribute to this. The in-depth knowledge of the properties of automated building processes and sustainable materials could ultimately help to transfer new architectural design concepts to the building construction industry and make them more sustainable.

## Figures and Tables

**Table 1:** Material specific results of the topology optimized geometries.

Process		MDF-Board	Epoxy Resin	ABS Plastic	Steel
Generative Design	Volume m^3^	2.95	3.78	3.47	3.37
	Mass Kg	117.65	215.54	184.15	1324.05
	Displacement cm	5	3	17.45	1
	Safety Factor	2.9	4.3	2	57.79

**Table 2:** Manufacturing settings and results of the TFP process.

Process	Parameter/Material	Value Material Type
TFP	Roving	Flax 1000tex
	Stitching Width	3 mm
	Stroke	10
	Machine Speed	500 stitches/min
	Base Material	Plain weave Glass, 105gsm HexForce 02,116 1260 TF970
	Sewing Thread	Amann Serafil 200/2
	Thread tension upper Thread	130 dN
	Thread tension under Thread	30 dN
	Needle	Grotz Beckert NM90
	Base Material	Plain weave glas 105 gsm

**Table 3:** Total and final amounts of resources used. ^1^ (p.P. = Per Preform).

Process	Resin/Curing Agent	Process Time (min)	Curing Time (h)	Volume (%)	Resin Used/Lost (~g/cm^3^)	Dry Weight (~g)
Vacuum Infusion	RIMR 135 (100 ± 2 g)	-	48 h	30 Fiber/70 Resin	165 p.P. ^1^	~218 p.P. ^1^
	RIMH 137 (30 ± 2 g)	300				
				1320 total	96 total	1744 total

**Table 4:** Total weight of assembled demonstrator.

Process	Elements	Flax Composite (g)
Demonstrator Scale 1:10	Branches	1.744
	Panels	2.535
	Total	4.279
